# Molecular characterization of *Blastocystis* sp., *Cryptosporidium* spp., and *Giardia* spp. in Eld’s deer (*Rucervus eldii*) and their forest rangers in Hainan, China

**DOI:** 10.1186/s13071-026-07434-w

**Published:** 2026-07-02

**Authors:** Yun Zhang, Jiaqi Li, Zhi Xie, Xican Han, Wanying Du, Yu Qiang, Guangxu Ren, Sheng Lei, Yunxing Chang, Xianrong Liu, Xiuyi Lai, Yuan Wang, Xinhui Li, Yunfei Zhou, Wentao Han, Xuming Qi, Hesheng Wang, Gang Lu

**Affiliations:** 1 Key Laboratory of Tropical Translational Medicine of Ministry of Education, Hainan Medical University-The University of Hong Kong Joint Laboratory of Tropical Infectious Diseases, Department of Pathogenic Biology, School of Basic Medicine Sciences, Hainan Medical University, Haikou, 571199 China; 2 Department of Nuclear Medicine, The 928, Hospital of PLA Joint Logistics Force, Haikou, 570100 China; 3 Bawangling Branch of Hainan Tropical Rainforest National Park Administration, Changjiang, 572700 China; 4 Hainan Bangxi Provincial Nature Reserve Administration, Baisha, 572821 China; 5 Hainan Datian National Nature Reserve Administration, Dongfang, 571129 China

**Keywords:** *Blastocystis* sp., *Cryptosporidium* spp., *Giardia* spp., Eld’s deer, Hainan

## Abstract

**Background:**

*Blastocystis* sp., *Cryptosporidium* spp., and *Giardia* spp. are significant zoonotic enteric protozoans colonizing/parasitizing humans and animals worldwide. Eld’s deer (*Rucervus eldii*) is a globally endangered tropical deer species. Prevalence and genetic diversity of these pathogens in wild Eld’s deer and occupationally exposed human populations remain limited. This study investigated the occurrence, molecular characteristics, and zoonotic potential of these three protozoans in wild Eld’s deer and their forest rangers in Hainan, China.

**Methods:**

A total of 217 fresh fecal samples were collected from wild Eld’s deer across two isolated habitats, and 19 stool samples were obtained from forest rangers, within the Hainan Bangxi Provincial Nature Reserve from March to August 2021. Genomic DNA was extracted and examined by polymerase chain reaction (PCR) targeting the SSU rRNA region for *Blastocystis* sp., and by nested PCR targeting the *SSU rRNA* region for *Cryptosporidium* spp., and the *bg* and *gdh* region for *Giardia* spp. The subtypes, species, and genotypes of positive amplicons were determined through sequence homology analysis and phylogenetic reconstruction using the neighbor-joining method.

**Results:**

The overall prevalence of *Blastocystis* sp., *Cryptosporidium* spp., and *Giardia* spp. in wild Eld’s deer were 25.8% (56/217), 5.5% (12/217), and 2.8% (6/217), respectively. Co-infections were detected in 3.2% (7/217) of deer samples. In Eld’s deer, six *Blastocystis* subtypes were identified (ST10, ST21, ST23, ST24, ST25, ST26), with ST10 being predominant (57.1%). Five *Cryptosporidium* species/genotypes were characterized (*Cryptosporidium bovis*, *Cryptosporidium xiaoi*, *Cryptosporidium* sp. bamboo rat genotype I, *Cryptosporidium* sp. bamboo rat genotype III, and *Cryptosporidium* sp. hamster genotype), with *C. bovis* being predominant (50.0%). All *Giardia* spp.-positive samples were identified as *Giardia bovis* (previously known as assemblage E). In the forest rangers, one *Blastocystis* ST3, two *C. xiaoi*, and one *Cryptosporidium* sp. hamster genotype were detected. Notably, the isolates of *Cryptosporidium* sp. hamster genotype in human and wild Eld’s deer showed 100% homology based on *SSU* ribosomal RNA (rRNA) gene sequencing analysis. Three novel sequences within *Blastocystis* subtypes (ST10 and ST26), three novel *Cryptosporidium* sequences (*Cryptosporidium* sp. bamboo rat genotype I, *Cryptosporidium* sp. bamboo rat genotype III, and *Cryptosporidium* sp. hamster genotype), and five novel *G. bovis* sequences (based on *bg* and *gdh* genes) were identified.

**Conclusions:**

This first molecular survey reveals high genetic diversity of *Blastocystis* sp., *Cryptosporidium* spp., and *Giardia* spp. in wild Eld’s deer in China, including the detection of shared *Cryptosporidium* species/genotypes between this endangered deer and sympatric forest rangers. These findings suggest potential zoonotic transmission at the wildlife–human interface and underscore the need for an integrated One Health surveillance strategy in protected areas to mitigate cross-species transmission risks, thereby contributing to both wildlife conservation and public health protection.

**Graphical Abstract:**

**Figure:**
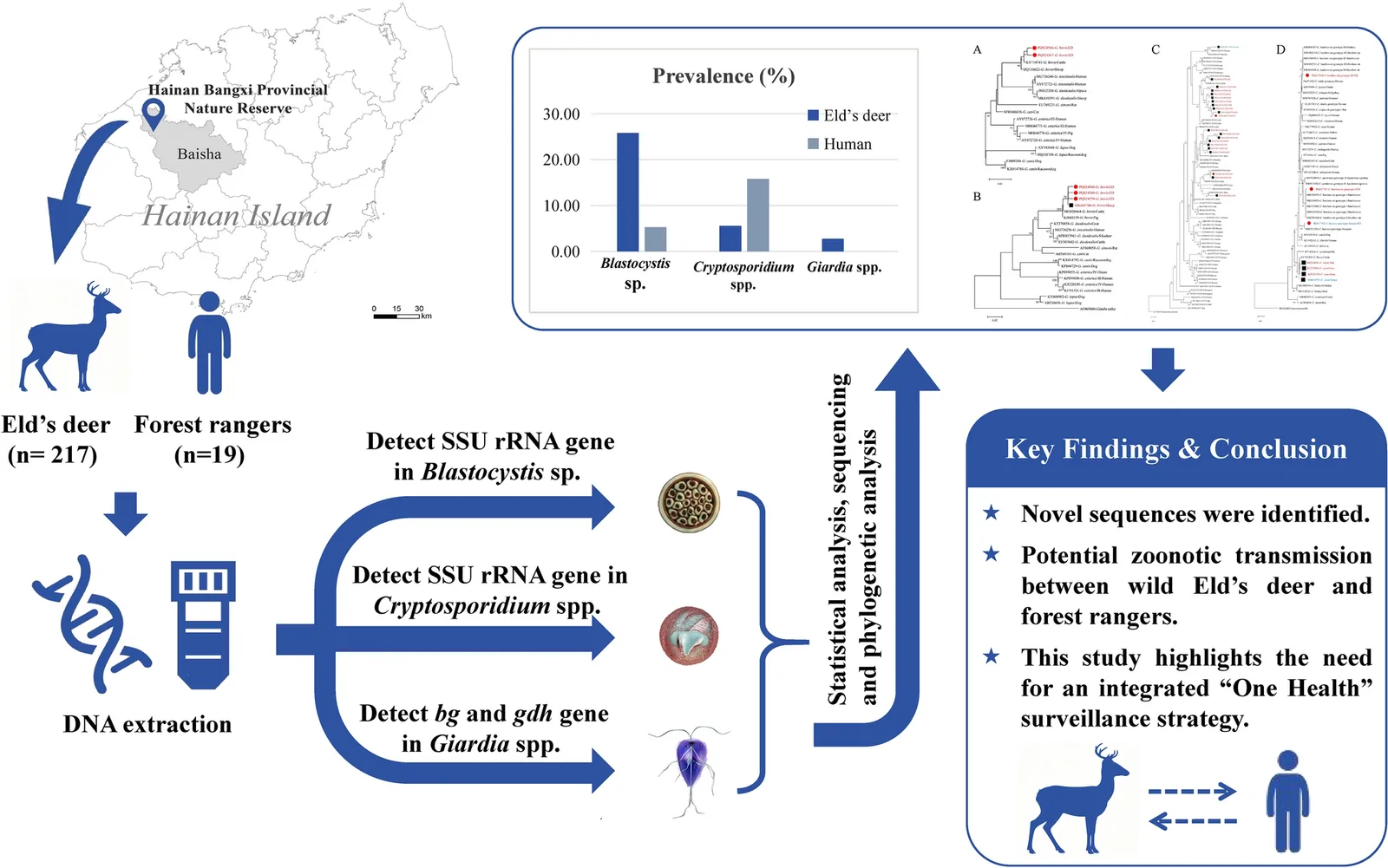

## Background

*Blastocystis* sp., *Cryptosporidium* spp., and *Giardia* spp. are important zoonotic enteric protozoans that can colonize/parasitize both human and nonhuman hosts worldwide [1, 2]. These pathogens are mainly transmitted via the fecal–oral route through the consumption of contaminated food and water, and are included as key reference pathogens in the Guidelines for Drinking-Water Quality (GDWQ) by the World Health Organization (WHO) [3]. They are associated with profuse or chronic diarrhea in humans and animals, including deer [4–6]. Wildlife is considered a potential source of *Blastocystis* sp., *Cryptosporidium* spp., and *Giardia* spp. [7, 8]. Recently, molecular epidemiological studies have identified these common enteric protozoans in captive and wild deer. To date, more than 18 *Blastocystis* subtypes (STs) have been identified in cervids, including ST1, ST2, ST3, ST4, ST5, ST10, and ST23, which have been reported in human [9–11]. More than ten species/genotypes of *Cryptosporidium* in deer have been detected, such as acknowledged zoonotic *Cryptosporidium hominis* and *Cryptosporidium parvum*; *Cryptosporidium xiaoi* (relatively rarely detected in humans); and those commonly prevalent in ungulates, *Cryptosporidium andersoni*, *Cryptosporidium bovis*, *Cryptosporidium ryanae*, *Cryptosporidium ubiquitum*, *Cryptosporidium muris*, *Cryptosporidium scrofarum*, *Cryptosporidium occultus*, and *Cryptosporidium* sp. deer genotype [12, 13]. Four *Giardia* species previously known as assemblages, including zoonotic *Giardia duodenalis* (A) and *Giardia enterica* (B), and animal-adapted *Giardia lupus* (D) and *Giardia bovis* (E), are occasionally found in cervids [13, 14].

Eld’s deer (*Rucervus eldii*) is a rare and globally endangered tropical deer species native to Southeast Asia, belonging to the order Artiodactyla, family Cervidae, and subfamily Cervinae. Due to illegal poaching and severe habitat encroachment, its global population has sharply declined [15]. The species is listed in Appendix I of the Convention on International Trade in Endangered Species of Wild Fauna and Flora (CITES), classified as Endangered on the International Union for Conservation of Nature (IUCN) Red List of Threatened Species, and designated as a Class I National Key Protected Wildlife species in China [16]. In China, the Eld’s deer is found only on Hainan Island. Due to rapid habitat destruction and intensive hunting, only 26 individuals remained in Hainan by the 1970s [17]. Currently, they primarily inhabit the Hainan Datian National Nature Reserve and the Hainan Bangxi Provincial Nature Reserve. Notably, the higher population density in the latter may increase the risk of disease outbreaks and the prevalence of intestinal pathogens among the deer [18]. However, little is known about the occurrence and genetic diversity of *Blastocystis* sp., *Cryptosporidium* spp., and *Giardia* spp. in wild Eld’s deer. This study investigated the molecular epidemiology and genetic characteristics of these enteric protozoans in wild Eld’s deer and their forest rangers in Hainan, China. Adopting a One Health perspective, these findings enhance our understanding of pathogen transmission at the wildlife–human interface and their ecological distribution while providing crucial baseline data for the conservation of this endangered species and for public health interventions in shared environments.

## Methods

### Sample collection

**Fig. 1:**
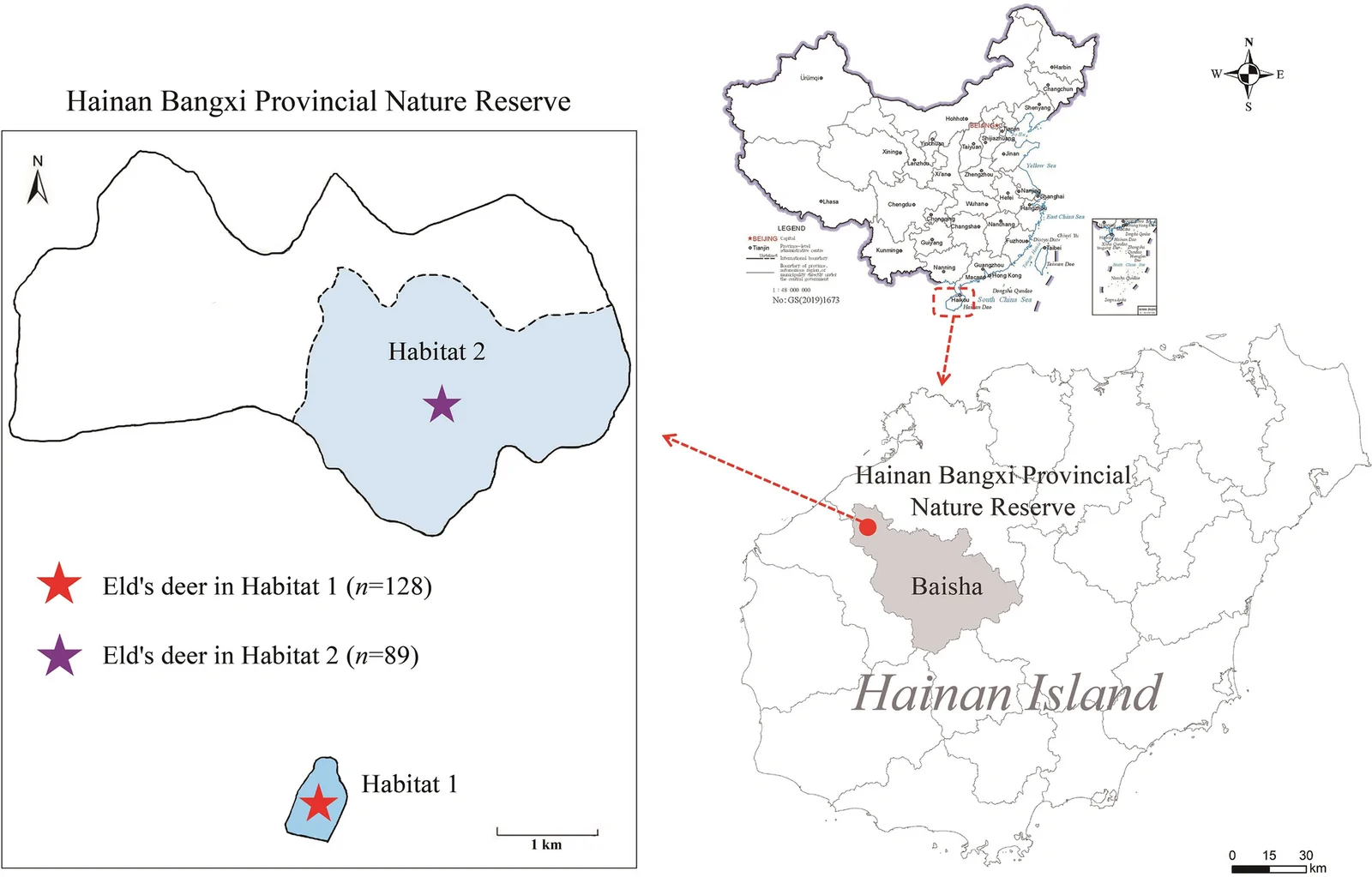
Map of sampling regions of Eld’s deer in the present study

From March to August 2021, a total of 217 fresh fecal samples were collected from wild Eld’s deer in two completely isolated areas of Hainan Bangxi Provincial Nature Reserve: Habitat 1 (*n* = 128) and Habitat 2 (*n* = 89) (Fig. 1). To ensure sample quality and minimize environmental contamination, the intact and untrampled fresh fecal samples were collected immediately after the deer were observed defecating using binoculars, with the assistance of experienced staff from the nature reserve, who then drove the deer away before collection. Specifically, approximately 20 g of fresh specimens were collected directly from the surface using sterile spoons, and only the inner portion of each specimen was taken to avoid contact with the underlying soil. The samples were then immediately placed into sterile 5 mL tubes. To ensure that samples were not collected from the same individual, each sample was taken at least 3 m apart. The samples were temporarily stored in a refrigerated insulated container. In parallel, fresh stool samples were also collected from forest rangers working in the Hainan Bangxi Provincial Nature Reserve (*n* = 19): 15 male and 4 female rangers, with ages ranging from 20 to 57 years. These rangers, who enter the reserve daily for patrols and represent a key population with potential contact with wild Eld’s deer, volunteered to participate in the study and were informed of the specific instructions for collecting the stool samples and preventing contamination. All 19 forest rangers were healthy individuals without any gastrointestinal symptoms at the time of sampling and voluntarily provided stool samples as part of the occupational health survey. All samples were transported to the laboratory and stored at −80 °C until analysis.

### DNA extraction and PCR amplification

Each stored fecal sample was washed three times with distilled water, followed each time by centrifugation at 3000 ×*g* for 10 min. Genomic DNA was then extracted directly from 200 mg of each processed fecal specimen using the QIAamp DNA Stool Mini Kit (Qiagen, Hilden, Germany), following the manufacturer’s instructions with an elevated lysis temperature of 95 °C to ensure a high DNA yield. The extracted DNA was stored at −20 °C until polymerase chain reaction (PCR) analysis.

PCR amplification of the extracted DNA was performed using genus-specific primers targeting the small subunit (SSU) *rRNA* gene of *Blastocystis* sp. and *Cryptosporidium* spp., and the β-giardin (*bg*) and glutamate dehydrogenase (*gdh*) genes of *Giardia* spp., following previously described protocols [19–23] (see Table 1 for primer details). After amplification, the PCR products were electrophoresed on a 1.5% agarose gel to identify positive amplicons, which were then purified using a QIAquick PCR Purification Kit (Qiagen, Hilden, Germany) according to the manufacturer’s instructions.

**Table 1:** Primers used in the study, annealing temperatures used in the PCR, and expected sizes of the PCR products

Genus	Gene	Primers sequence(5′-3′)	Annealing temperature (℃)	Amplicon length (bp)	References
*Blastocystis* sp.	*SSU rRNA*	Forward primer RD1: 5′-GGAGGTAGTGACAATAAATC-3′ Reverse primer RD2: 5′-TGCTTTCGCACTTGTTCATC-3′	54	~500	[19]
*Cryptosporidium* spp.	*SSU rRNA*	The first amplification: Forward primer SHP1: 5′-ACCTATCAGCTTTAGACGGTAGGGTAT-3′ Reverse primer SHP2: 5′-TTCTCATAAGGTGCTGAAGGAGTA AGG-3′	56	~600	[20]
		The second amplification: Forward primer SHP3: 5′-ACAGGGAGGTAGTGACAAGAAATAACA-3′ Reverse primer SHP4: 5′-AAGGAGTAAGGAACAACCTCCA-3′	56		
*Giardia* spp.	*bg*	The first amplification: Forward primer G7: 5′-AAGCCCGACGACCTCACCCGCAGTGC-3′ Reverse primer G759: 5′-GAGGCCGCCCTGGATCTTCGAGACGAC-3′	65	~511	[21]
		The second amplification: Forward primer F2: 5′-GAACGAACGAGATCGAGGTCCG-3′ Reverse primer R2: 5′-CTCGACGAGCTTCGTGTT-3′	55		[22]
	*gdh*	External forward primer GDHeF: 5′-TCAACGTYAAYCGYGGYTTCCGT-3′ Internal forward primer GDHiF: 5′-CAGTACAACTCYGCTCTCGG-3′ Reverse primer GDHiR: 5′-GTTRTCCTTGCACATCTCC-3′	56	~432	[23]

### Sequencing and phylogenetic analysis

All positive PCR products were purified prior to sequencing. The purified products were then sequenced in both directions using the BigDye Terminator v3.1 Cycle Sequencing Kit (Applied Biosystems, USA) and an ABI PRISM 3730 XL DNA Analyzer (Thermo Fisher Scientific, Waltham, MA, USA). Sequence accuracy was confirmed by bidirectional sequencing. The obtained sequences were aligned with each other and compared with reference sequences downloaded from GenBank using the Basic Local Alignment Search Tool (BLAST) (http://www.ncbi.nlm.nih.gov/BLAST/) and ClustalX 1.83 (http://www.clustal.org/) to determine the species or genotypes represented. Phylogenetic analyses were performed using the *SSU rRNA* gene sequences of *Blastocystis* sp. and *Cryptosporidium* spp., and the *bg* and *gdh* gene sequences of *Giardia* spp. obtained in this study, along with representative sequences available in the GenBank database. Phylogenetic trees were constructed using Molecular Evolutionary Genetics Analysis, Version 11.0 (MEGA 11) [24] with the neighbor-joining (NJ) method under the Kimura 2-parameter model, supported by 1000 bootstrap replicates.

The representative nucleotide sequences obtained in this study have been deposited in the GenBank database under the following accession numbers: ON261379, ON261518, and ON261403 for *Blastocystis* sp.; PQ817700–PQ817702 for *Cryptosporidium* spp.; and PQ824566–PQ824570 for *Giardia* spp.

### Statistical analysis

Statistical analysis was performed using the Statistical Package for the Social Sciences (SPSS) version 25.0 (SPSS Inc., Chicago, IL, USA). Chi square analysis was performed to compare the prevalence of *Blastocystis* sp., *Cryptosporidium* spp., and *Giardia* spp. in Eld’s deer between different habitats. Differences were considered statistically significant when *P* < 0.05. In the analysis of the prevalence, if more than 20% of the cells have an expected value of less than 5, Fisher’s exact test was used. Multilevel logistic mixed regression models were constructed to calculate 95% confidence intervals (CI).

## Results

### Prevalence and coinfection of enteric protozoans

A total of 217 fecal specimens from Eld’s deer and 19 samples from humans were analyzed using PCR assays targeting *Blastocystis* sp., and nested PCR for *Cryptosporidium* spp. and *Giardia* spp. The most prevalent protozoan parasite detected in Eld’s deer was *Blastocystis* sp., with an overall prevalence of 25.8% (56/217, 95% CI: 19.98–31.63), followed by *Cryptosporidium* spp. (5.5%, 12/217, 95% CI: 2.49–8.57) and *Giardia* spp. (2.8%, 6/217, 95% CI: 0.58–4.95). Specifically, the infection rates of *Blastocystis* sp. and *Giardia* spp. in Eld’s deer from Habitat 1 were higher than those from Habitat 2. In contrast, *Cryptosporidium* spp. infection rates showed the opposite trend (Table 2). No significant differences were observed in the infection rates of these protozoans in Eld’s deer between the two completely independent habitats under investigation (*P* > 0.05). In the present survey, we detected co-infections with multiple enteric protozoans in seven (3.2%) Eld’s deer fecal samples. These comprised four cases of co-infection between *Blastocystis* sp. and *Giardia* spp. (1.8%) and three cases of co-infection between *Blastocystis* sp. and *Cryptosporidium* spp. (1.4%). A higher prevalence of mixed intestinal protozoan infections was observed in Eld’s deer from Habitat 1 (5.6%) compared with those from Habitat 2 (1.6%) (*P* > 0.05 in Fisher’s exact test) (Table 2).

**Table 2:** Prevalence and distribution of *Blastocystis* sp., *Cryptosporidium* spp., and *Giardia* spp. genotypes/subtypes in Eld’s deer and forest rangers in Hainan, China

Pathogens	Eld’s deer	Prevalence (%) (number positive/number examined) (95% CI)	*P*-value	Genotypes/subtypes (*n*)	Human	Prevalence (%) (number positive/number examined) (95% CI)	*P*-value	Genotypes/subtypes (*n*)
*Blastocystis* sp.	Habitat 1	31.5 (28/89) (21.81–41.11)	*P* = 0.112^a^	ST10 (21), ST25 (5), ST26 (2)	Male	0 (0/14)	*P* = 0.053^b^	—
	Habitat 2	21.9 (28/128) (14.71–29.04)		ST24 (13), ST10 (11), ST21 (2), ST23 (1), ST26 (1)	Female	20.0 (1/5) (−15.06–55.06)		ST3 (1)
	Total	25.8 (56/217) (19.98–31.63)		ST10 (32), ST24 (13), ST25 (5), ST26 (3), ST21 (2), ST23 (1)	Total	5.3 (1/19) (−4.78 to 15.30)		ST3 (1)
*Cryptosporidium* spp.	Habitat 1	3.4 (3/89) (−0.38 to 7.12)	*P* = 0.367^b^	*C. xiaoi* (3)	Male	14.3 (2/14) (−4.04 to 32.62)	*P* = 0.158^b^	*C. xiaoi* (1), *C. hamster* genotype (1)
	Habitat 2	7.0 (9/128) (2.60–11.46)		*C. bovis* (6), *C*. bamboo rat genotype I (1), *C*. bamboo rat genotype III (1), *C. hamster* genotype (1)	Female	20.0 (1/5) (−15.06 to 55.06)		*C. xiaoi* (1)
	Total	5.5 (12/217) (2.49–8.57)		*C. bovis* (6), *C. xiaoi* (3), *C*. bamboo rat genotype I (1), *C.* bamboo rat genotype III (1), *C. hamster* genotype (1)	Total	15.8 (3/19) (−0.61 to 32.19)		*C. xiaoi* (2), *C. hamster* genotype (1)
*Giardia* spp.	Habitat 1	4.5 (4/89) (0.19–8.80)	*P* = 0.230^b^	*G. bovis* (4)	Male	0 (0/14)		—
	Habitat 2	1.6 (2/128) (−0.59 to 3.71)		*G. bovis* (2)	Female	0 (0/5)		—
	Total	2.8 (6/217) (0.58–4.95)		*G. bovis* (6)	Total	0 (0/19)		—
Co-infections	Habitat 1	5.6 (5/89) (0.83–10.40)	*P* = 0.126^b^	ST10+ *G. bovis* (3), ST10+ *C. xiaoi* (1), ST25+ *C. xiaoi* (1)	Male	0 (0/14)		—
	Habitat 2	1.6 (2/128) (−0.59 to 3.17)		ST24+ *G. bovis* (1), ST10+ *C*. bamboo rat genotype I (1)	Female	0 (0/5)		—
	Total	3.2 (7/217) (0.87–5.58)		ST10+ *G. bovis* (3), ST10+ *C. xiaoi* (1), ST25+ *C. xiaoi* (1), ST24+ *G. bovis* (1), ST10+ *C*. bamboo rat genotype I (1)	Total	0 (0/19)		—

Em-dash (—) indicates “not detected.”

^a^*P*-value in the Chi square test

^b^*P*-value in the Fisher’s exact test

Furthermore, the overall prevalence of *Blastocystis* sp. and *Cryptosporidium* spp. in forest rangers working in the nature reserve was 5.3% (1/19, 95% CI: −4.78 to 15.30) and 15.8% (3/19, 95% CI: −0.61 to 32.19), respectively. *Giardia* spp. were not detected in humans (Table 2). Due to the small sample size of forest rangers in this study, protozoan infection rates were not analyzed by gender. None of the human participants exhibited co-infections with multiple intestinal protozoan species.

### Molecular characterization of enteric protozoans

According to the *SSU rRNA* gene sequence analysis, six *Blastocystis* subtypes in Eld’s deer were identified in present study, with ST10 being the predominant subtype (*n* = 32), followed by ST24 (*n* = 13), ST25 (*n* = 5), ST26 (*n* = 3), ST21 (*n* = 2), and ST23 (*n* = 1). The diversity of *Blastocystis* subtypes in Eld’s deer from Habitat 2 was higher than that in Habitat 1. Both ST10 and ST26 were found in Eld’s deer from both habitats, while ST25 was detected only in Habitat 1. Conversely, ST24, ST21, and ST23 were exclusively identified in Eld’s deer from Habitat 2 (Table 2). Only one *Blastocystis* isolate was identified as ST3 in a forest ranger (Table 2).

Sequence analysis of the *SSU rRNA* gene revealed the presence of five *Cryptosporidium* species/genotypes in Eld’s deer, namely *C. bovis* (*n* = 6), *C. xiaoi* (*n* = 3), *Cryptosporidium* sp. bamboo rat genotype III (*n* = 1), *Cryptosporidium* sp. bamboo rat genotype I (*n* = 1), and the *Cryptosporidium* sp. hamster genotype (*n* = 1). The *Cryptosporidium* species identified in Eld’s deer from the two completely isolated habitats were entirely distinct, with higher species diversity observed in Habitat 2. In Habitat 1, three *C. xiaoi* isolates were detected, whereas four *Cryptosporidium* species were identified in Habitat 2, with *C. bovis* being the dominant species (Table 2). Additionally, three *Cryptosporidium* isolates were detected in forest rangers, including two *C. xiaoi* (from one male and one female) and one *Cryptosporidium* sp. hamster genotype (from another male) (Table 2). Notably, the *Cryptosporidium* sp. hamster genotype sequences detected in forest rangers and wild Eld’s deer showed 100% homology.

For *Giardia* spp., sequence analysis of the *bg* (*n* = 2) and *gdh* (*n* = 4) genes revealed that all six positive samples from Eld’s deer belonged to *G. bovis*. Additionally, *Giardia* spp. were not detected in humans in this study (Table 2).

### Phylogenetic analysis of enteric protozoans

**Fig. 2:**
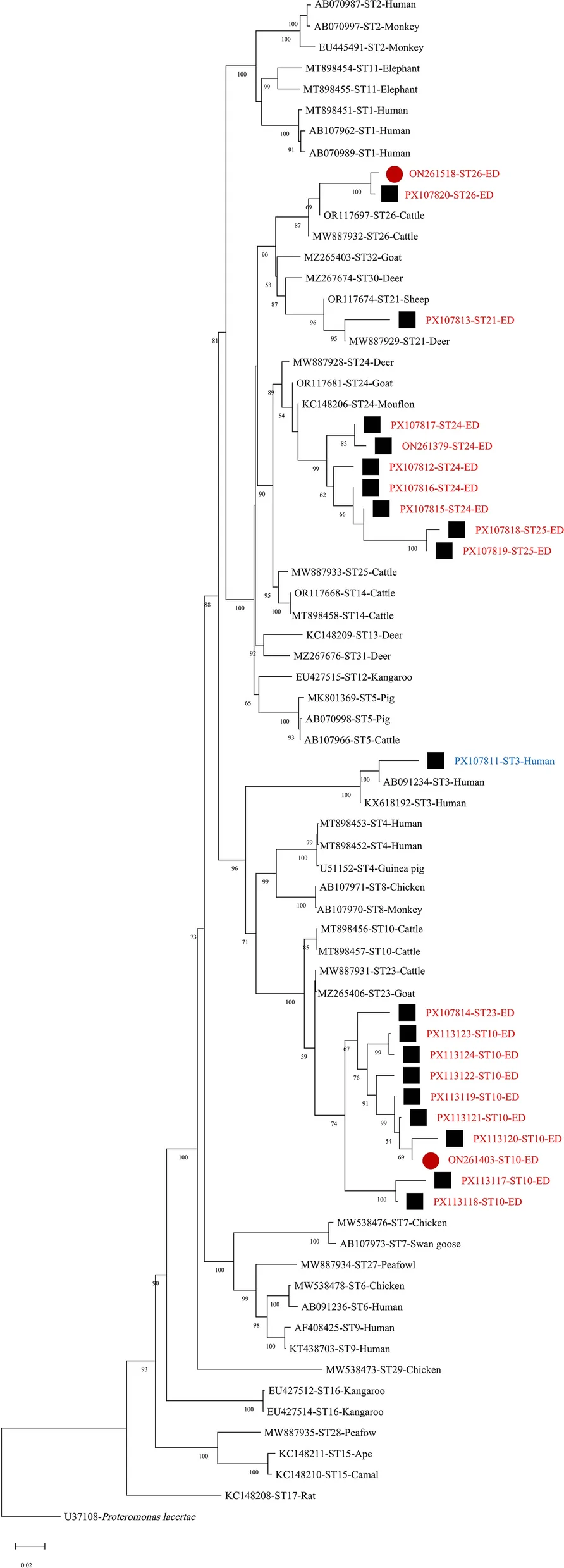
Phylogenetic relationships of Blastocystis sp. from Eld’s deer and their forest rangers based on the *SSU rRNA* gene by Neighbor-Joining (NJ) analysis using general time-reversible model. The known and novel sequences identified in this study were indicated by black squares and red circles, respectively. Blue text indicates that the sequence of the isolate comes from humans, while red text indicates that sequence of the isolate comes from Eld’s deer. The sequence of *Proteromonas lacertae* (U37108) was as an out-group

**Fig. 3:**
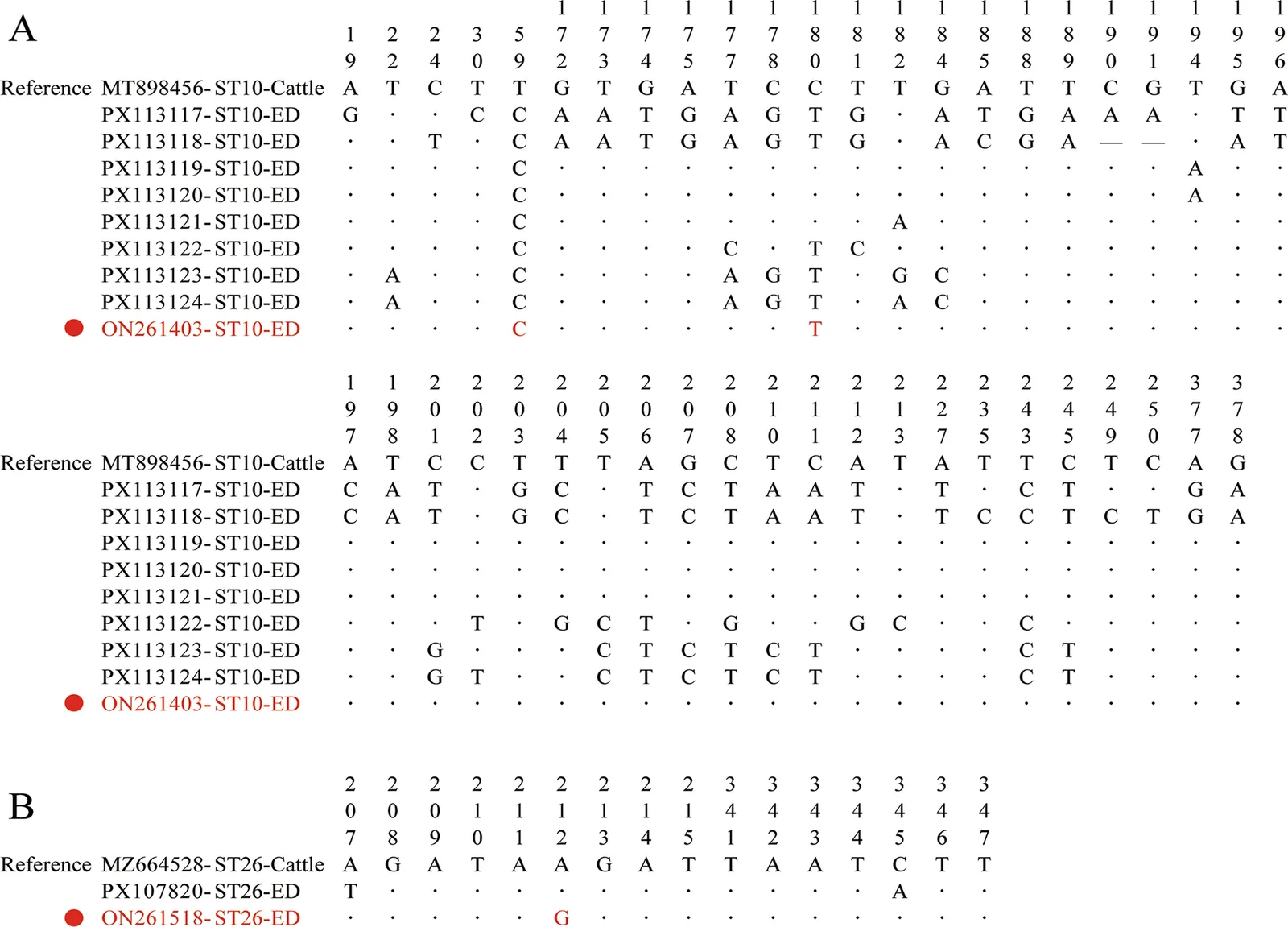
The *SSU rRNA* gene sequence polymorphism among novel and known sequences of ST10 (**A**) and ST26 (**B**) with the reference sequence. The novel sequences identified in this study were indicated by red circles

Phylogenetic analysis using the neighbor-joining (NJ) method showed that the representative *Blastocystis* subtype sequences obtained in this study clustered with the reference sequences in their corresponding subtype branches (Fig. 2). A nucleotide sequence was considered novel if it exhibited at least one nucleotide substitution, deletion, or insertion compared with known subtype sequences. A novel sequence that differs by at least 3% from other sequences within a known clade may be defined as a new subtype [25]. In addition to 60 known sequences (ST10, *n* = 31; ST24, *n* = 13; ST25, *n* = 5; ST26, *n* = 1; and ST23, *n* = 1) identified in Eld’s deer and forest rangers, three novel sequences were identified in *Blastocystis* isolates from Eld’s deer: one novel ST10 (ON261403) and two novel ST26 (ON261518). The novel ST10 sequence (ON261403) showed 99.75% identity to MK244919 isolated from cattle in the USA [26] and exhibited two nucleotide substitutions at positions 59 (T → C) and 180 (C → T) (Fig. 3A). The novel ST26 sequence (ON261518) showed 99.79% identity to MZ664528 isolated from cattle in Spain [27] and exhibited a nucleotide substitution at position 212 (A → G) (Fig. 3B).

**Fig. 4:**
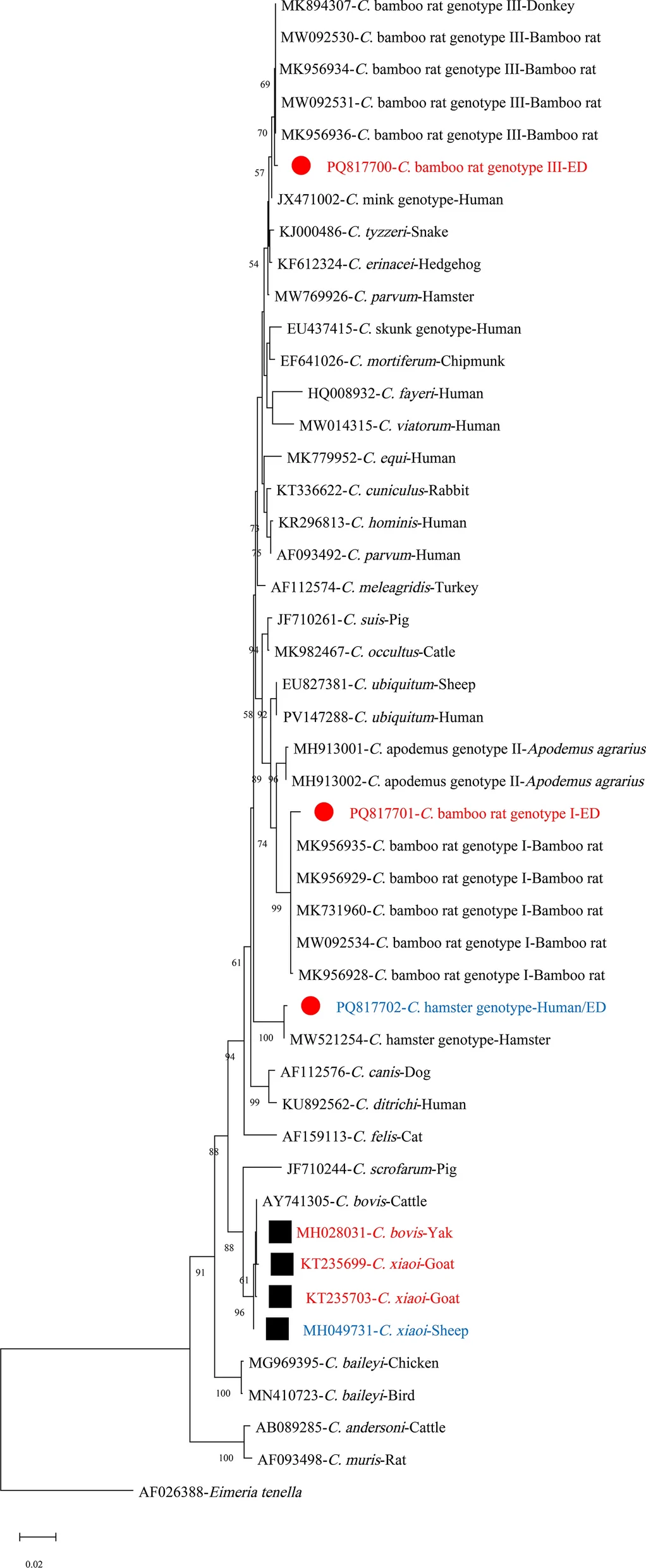
Phylogenetic relationships of *Cryptosporidium* spp. from Eld’s deer and their forest rangers based on the *SSU rRNA* gene by neighbor-joining (NJ) analysis using a general time-reversible model. The known and novel sequences identified in this study were indicated by black squares and red circles, respectively. Blue text indicates that the sequence of the isolate comes from humans, while red text indicates that sequence of the isolate comes from Eld’s deer. The sequence of *Eimeria tenella* (AF026388) was as an out-group

**Fig. 5:**
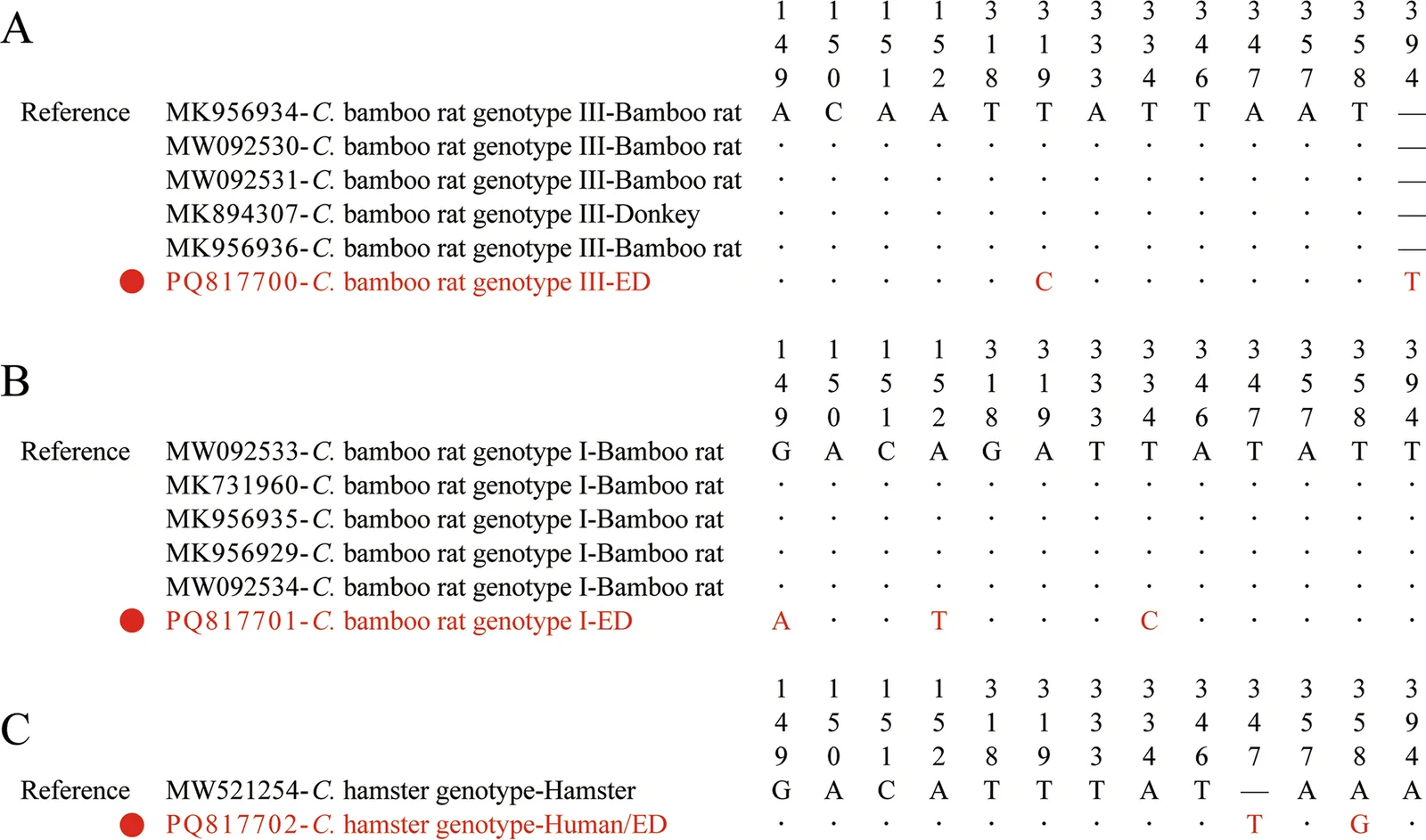
The *SSU rRNA* gene sequence polymorphism among novel sequences of *Cryptosporidium* sp. bamboo rat genotype III, *Cryptosporidium* sp. bamboo rat genotype I, and *Cryptosporidium* sp. hamster genotype with the reference sequence. The novel sequences identified in this study were indicated by red circles

All the sequences of *Cryptosporidium* obtained in our study were 568 bp fragments, which target a conserved region that is widely accepted and routinely used in molecular epidemiological studies for *Cryptosporidium* species/genotype identification. While the fragment is shorter than the full‑length *SSU rRNA* gene, it is sufficient for phylogenetic placement and species/genotype assignment. Phylogenetic analysis based on the *SSU rRNA* gene of *Cryptosporidium* spp. revealed species diversity within *C. bovis*, *C. xiaoi*, *Cryptosporidium* sp. bamboo rat genotype III, *Cryptosporidium* sp. bamboo rat genotype I, and *Cryptosporidium* sp. hamster genotype in Eld’s deer and forest rangers in the present study (Fig. 4). The novel *Cryptosporidium* sp. bamboo rat genotype III sequence (PQ817700) from Eld’s deer showed 99.65% identity to several sequences isolated from a donkey and bamboo rats in China (MK894307, MK956934, MK956936, MW092530, and MW092531) [28, 29], and exhibited two nucleotide substitutions: at positions 319 (T → C) and 394 (deletion → T). The novel *Cryptosporidium* sp. bamboo rat genotype I sequence (PQ817701) from Eld’s deer showed 99.48% identity to several sequences isolated from bamboo rats in China (MK956929, MW092533, MW092534, MK731960, MK956935) [28–30] and exhibited three nucleotide substitutions: at positions 149 (G → A), 152 (A → T), and 334 (T → C). The sequences MW092530 and MW092533 were selected for single nucleotide polymorphisms (SNPs) analysis of the novel sequences PQ817700 and PQ817701, respectively (Fig. 5). The novel *Cryptosporidium* sp. hamster genotype sequence (PQ817702), identified in both an Eld’s deer and a forest ranger, showed 99.74% identity to MW521254 isolated from a hamster in China [31] and exhibited two nucleotide substitutions at positions 347 (deletion → T) and 358 (A → G) (Fig. 5). The six *C. bovis* isolates identified in Eld’s deer were 100% homologous to a known *C. bovis* isolate from a yak in China (MH028031). The three *C. xiaoi* isolates identified in Eld’s deer displayed 100% identity to three *C. xiaoi* isolates from goats in China (KT235699, *n* = 2; KT235703, *n* = 1) [32]. The two *C. xiaoi* isolates identified in forest rangers were 100% homologous to a known *C. xiaoi* isolate from a sheep in China (MH049731).

**Fig. 6:**
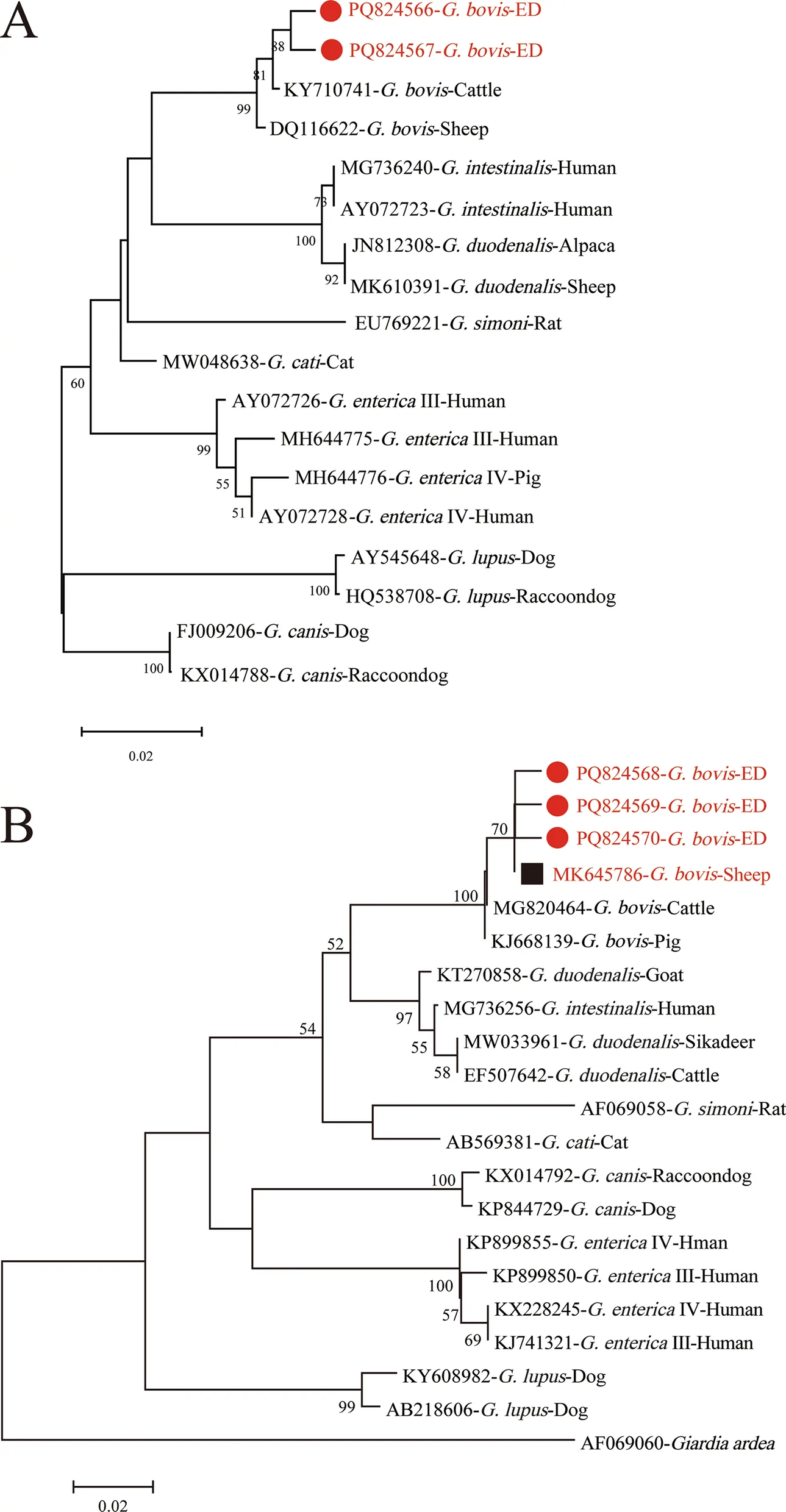
Phylogenetic relationships of *Giardia* spp. from Eld’s deer based on the *bg* gene (**A**) and *gdh* gene (**B**) by neighbor-joining (NJ) analysis using a general time-reversible model. The known and novel sequences identified in this study were indicated by black squares and red circles, respectively. The sequence of *Giardia ardea* (AF069060) was as an out-group

**Fig. 7:**
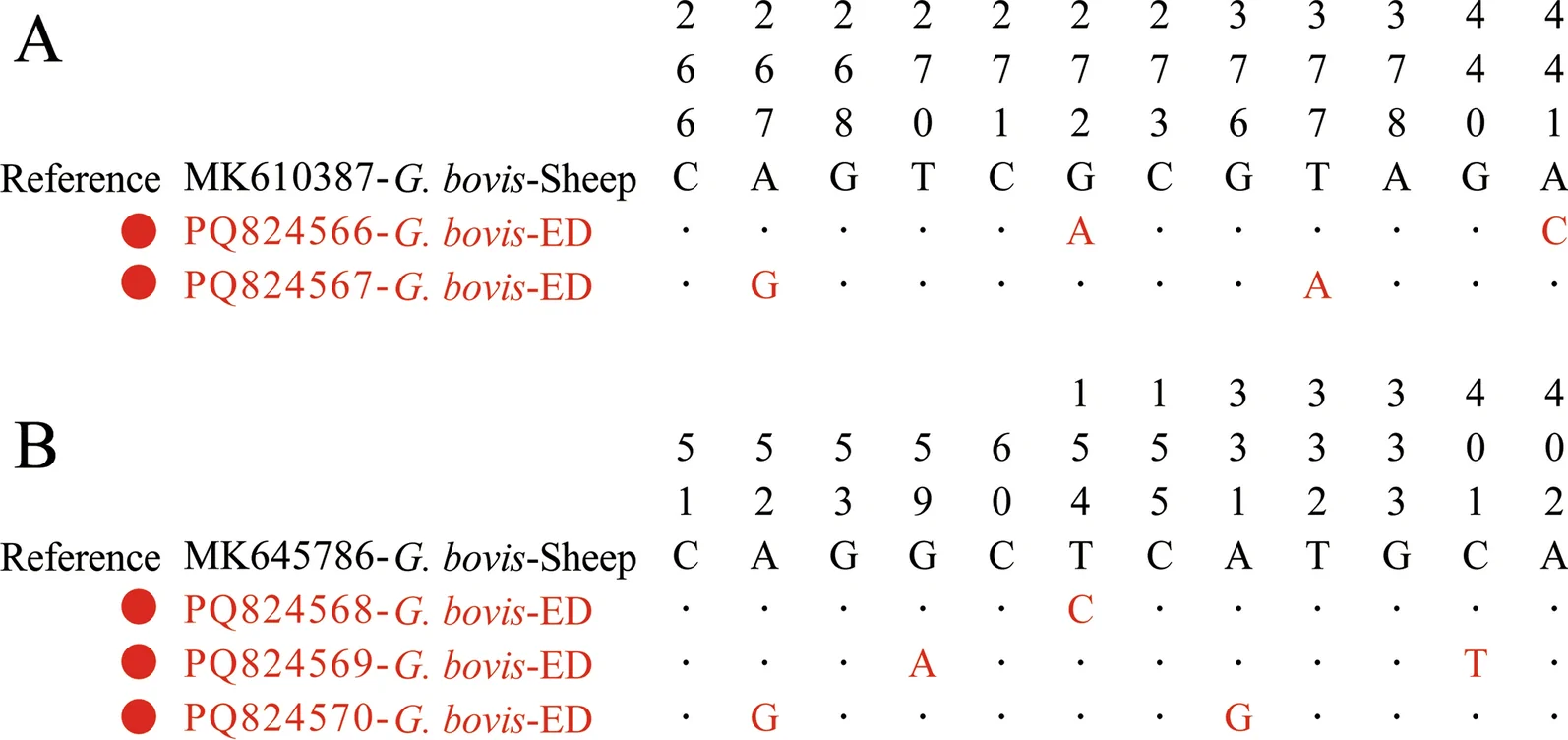
The *bg* gene (**A**) and *gdh* gene (**B**) sequence polymorphism among novel and known sequences of *Giardia* spp. species with the reference sequence. The novel sequences identified in this study were indicated by red circles

The results of the phylogenetic analysis based on the *bg* gene and *gdh* gene by the NJ method showed consistent clustering patterns for *Giardia* spp. in Eld’s deer, and all the six obtained sequences of this study clustered with the reference sequences in their corresponding *G. bovis* branches (Fig. 6A, B). Two novel *G. bovis* sequences (PQ824566 and PQ824567) from Eld’s deer were identified and showed 99.61% identity with MK610387 [33], a sequence isolated from sheep in China based on the *bg* gene. Compared with the reference sequence, the PQ824566 sequence has two nucleotide substitutions at position 272 (G → A) and 441 (A → C), while the PQ824567 sequence has 2 nucleotide substitutions at position 267 (A → G) and 377 (T → A). In addition to a known *G. bovis* sequence (MK645786) from Chinese sheep [32] based on the *gdh* gene, three novel sequences (PQ824568–PQ824570) were identified. These novel sequences shared 99.58–99.79% identity with MK645786, and the PQ824568 sequence has two nucleotide substitutions at position 52 (A → G) and 331 (A → G), the PQ824570 sequence has two nucleotide substitutions at position 59 (G → A) and 401 (C → T), while the PQ824569 sequence only has a nucleotide substitution at position 154 (T → A) (Fig. 7).

## Discussion

Cervids can serve as reservoirs for important pathogens of livestock and humans [34]. To the best of our knowledge, this study represents the first report of *Blastocystis* sp., *Cryptosporidium* spp., and *Giardia* spp. in Eld’s deer worldwide, as well as their prevalence in personnel working within the Eld’s deer nature reserve. The prevalence of *Blastocystis* sp. in wild Eld’s deer in Hainan was 25.8% (56/217) (Table 2), which is similar to that reported in wild red deer (*Cervus elaphus*) from Australia (27.1%, 19/70) [35]; free-ranging Père David’s deer (*Elaphurus davidianus*) from Beijing, China (29.0%, 83/286) [8]; and captive fallow deer (*Dama dama*) from China (30.8%, 4/13) [36]. However, it was higher than that in wild red deer from Australia (2.0%, 1/50) [37]; wild fallow deer (4.2%, 4/96), red deer (11.9%, 39/329) and roe deer (*Capreolus capreolus*) (12.9%, 12/93) from Spain [38]; wild white-lipped deer (*Cervus albirostris*) from Qinghai (12.5%, 1/8) [39]; and forest musk deer (*Moschus berezovskii*) from Sichuan, China (14.7%, 74/504) [11]. In contrast, higher positive rates of *Blastocystis* sp. have been reported in wild white-tailed deer (*Odocoileus virginianus*) (88.8%, 71/80) from the USA [40], wild Père David’s deer (56.3%, 72/128) [9] and captive sika deer (*Cervus nippon*) (65.0%, 303/466) [10] from northern China, wild fallow deer (63.3%, 31/49) and sambar deer (*Rusa unicolor*) (48.2%, 192/398) [35] from Australia, wild sika deer from Japan (45.5%, 60/132) [41], wild Korean water deer (*Hydropotes inermis argyropus*) (40.8%, 51/125) from Korea [42], and wild red deer from Portugal (40.4%, 38/94) [38]. Previous meta-analyses have reported a worldwide prevalence of *Cryptosporidium* spp. in ten deer species ranging from 1.1% (4/298) to 62.5% (20/32) [43]. The prevalence rate in Asian deer (5.5%, 162/3501) [43] is similar to the 5.5% (12/217) found in wild Eld’s deer in Hainan in our study (Table 2). *Cryptosporidium* spp. have been reported at highly variable prevalence rates in wild deer populations: in fallow deer (33.3%, 70/210), roe deer (17.2%, 30/174), and red deer (9.7%, 6/62) in Germany [12]; in wild white-tailed deer (12.5%, 10/80) in the USA [44]; and in captive sika deer (14.8–15.1%) in northern China [45, 46]. Additionally, the prevalence rates reported in other studies were lower than that in our study, including those in wild Père David’s deer (1.5–1.6%) in China [5, 47]; wild red deer in Italy (0.5%, 1/196) [48], Spain (1.5%, 10/653) [49], and Portugal (3.1%, 3/96) [50]; wild roe deer in Italy (3.4%, 4/118) [50]; and captive spotted deer (*Axis axis*) in Bangladesh (3.3%, 1/30) [51]. In the present study, the prevalence of *Giardia* spp. in wild Eld’s deer was 2.8% (6/217). This rate is similar to prevalence found in captive cervid species from other studies, including forest musk deer (2.2%, 5/223) in China [52] and spotted deer (3.3%, 1/30) in Bangladesh [51]. To date, high prevalence rates of *Giardia* spp. infections have been reported in cervids in several countries, including in wild fallow deer (5.2–11.5%) in Spain and Italy [49, 53]; wild red deer (3.8–17.9%) and roe deer (7.5–22.9%) in Spain, Portugal, and Norway [49, 50, 54–56]; and captive alpine musk deer (*Moschus chrysogaster*) (19.3%, 39/202) in China [57]. Furthermore, lower infection rates have been reported: 1.3% (1/80) in wild white-tailed deer from the USA [44], 1.1% (4/374) in wild red deer from Croatia [58], 0.7% (2/271) in wild sika deer from Japan [59], and 0.6% (5/818) in a captive population in northeast China [14]. The differences in the prevalence of *Blastocystis* sp., *Cryptosporidium* spp., and *Giardia* spp. in deer are likely attributable to variations in animal species and breeds, detection methods, sample sizes, geographic regions, and management practices [60, 61].

A recent study conducted in a tropical wildlife park in Hainan Province reported the occurrence of the same intestinal protozoa in 28 animal species. It was reported that ST10 and ST5 were the dominant *Blastocystis* subtypes from animals in this park. ST10 was identified in free-ranging white buffalo, sika deer, macaques, and Eld’s deer, and ST5 was detected in alpacas, Eld’s deer, emus, red-bellied squirrels, beaver rats, and guinea pigs. *Cryptosporidium ubiquitum* (OQ993216) and *Cryptosporidium* sp. bamboo rat genotype I (OQ993215) were identified in different animal hosts in the park (sika deer, emus, and beaver rats) being the predominant *Cryptosporidium* species/genotype, followed by *C. xiaoi* (OQ993214) which was obtained in white buffalo and Père David’s deer. One *Cryptosporidium* sp. bamboo rat genotype III isolate (OQ993217), previously known as *C. parvum*-like, was detected in an African lion. *Giardia bovis* (previously known as assemblage E) was the only species in sika deer and Eld’s deer in the park, providing a valuable opportunity for regional comparison [62]. In our study, based on *SSU rRNA* gene sequence analysis, six subtypes of *Blastocystis* sp. (ST10, ST21, ST23, ST24, ST25, and ST26) were identified in 56 positive wild Eld’s deer samples. ST10 was the predominant subtype in Eld’s deer, consistent with the detection results in animals from the tropical wildlife park in Hainan [62], further supporting its established role as the primary subtype in artiodactyls, including pigs, cattle, goats [63], sheep [64], camelids [65], and deer [11, 40]. ST21 and ST23–26, which were also detected in Eld’s deer, have been reported in various other deer species (Père David’s deer [9], sika deer [10], white-tailed deer [40], Korean water deer [42], and musk deer [66]) and appear to be prevalent in ruminants [38, 67–69]. While ST10 was initially considered primarily herbivore-associated with minimal reported human infections [70], it has since been documented in human populations in Egypt [71], Senegal [72], Vietnam [73], and Thailand [74]. Similarly, ST23 was identified as the dominant subtype in 12 human cases in Thailand [74], indicating its zoonotic potential and public health significance. Additionally, ST21, ST25, and ST26 are classified as non-zoonotic subtypes [75], with no reported cases of human infection. Notably, one ST3-positive sample from a forest ranger was detected in this study. ST3 has been reported as the most predominant subtype among school children in Hainan [76], as well as in humans in Iran, Türkiye [77], Azerbaijan, Jordan, and Tanzania [78]. It has also been frequently detected in water sources [79] and animals [40, 80], suggesting the potential for reverse zoonotic transmission between humans and Eld’s deer. Based on the sequence alignment, notable genetic polymorphisms were observed among *Blastocystis* ST10 isolates from wild Eld’s deer. Multiple SNPs and deletion events were detected at specific loci in these samples. This diversity may be attributed to host adaptation, geographical separation, or underlying evolutionary pressures.

Sequence analysis revealed five *Cryptosporidium* species/genotypes in wild Eld’s deer (*C. bovis*, *C. xiaoi*, *Cryptosporidium* sp. bamboo rat genotype I, *Cryptosporidium* sp. bamboo rat genotype III, and *Cryptosporidium* sp. hamster genotype) and two in forest rangers working in the nature reserve (*C. xiaoi* and *Cryptosporidium* sp. hamster genotype). *Cryptosporidium bovis* is the dominant species in Eld’s deer, a species that has also been reported in roe deer in Spain [54] and sika deer in Japan [59] and China [60]. Moreover, it is the prevalent in other cloven-hoofed animals, such as cattle [54, 81, 82], yaks [83], and sheep [84]. Although *C. bovis* is occasionally reported in neonatal calves, its true clinical and zoonotic significance has been largely overlooked [85]. In this study, one *Cryptosporidium* sp. bamboo rat genotype I isolate and one *Cryptosporidium* sp. bamboo rat genotype III isolate were detected in wild Eld’s deer. *Cryptosporidium ubiquitum* has been detected in cervids in various countries, including red deer in the Czech Republic [86], roe deer in Italy [48], white-tailed deer in the USA [87], swamp deer (*Cervus duvaucelii*) in Nepal [88], deer (sambar deer, red deer, and fallow deer) in Australia [89], and Père David’s deer, farmed sika deer, and reindeer in China [13, 46]. In contrast, *Cryptosporidium* sp. bamboo rat genotype I, although phylogenetically related to *C. ubiquitum*, has not yet been identified in deer. The sequence of *Cryptosporidium* sp. bamboo rat genotype III was originally described in donkeys in China and named *Cryptosporidium* mink genotype-like [90]. This genotype was subsequently named the *C. parvum*-like genotype when it was identified in farmed bamboo rats in China [28] and has recently been redescribed as *Cryptosporidium* bamboo rat genotype III [29]. In our study, *Cryptosporidium* sp. bamboo rat genotype III was identified in cervids for the first time, which expands its host range and suggests its potential for cross-species transmission. *Cryptosporidium xiaoi* was identified in three wild Eld’s deer and two forest rangers working in the nature reserve. It has also been detected in reindeer (*Rangifer tarandus*) in northeast China [46] and alpine musk deer in northwest China [91]. This species is the most commonly reported *Cryptosporidium* species in sheep and goats in Ghana [92], South Korea [93], and Iran [94], as well as in Inner Mongolia, Xinjiang, Heilongjiang, and Qinghai in China [68, 94–98], and is potentially responsible for caprine cryptosporidiosis [93]. Although *C. xiaoi* was generally not considered to infect humans and was not described as zoonotic [99], it has been identified in patients human immunodeficiency virus(HIV)/acquired immunodeficiency syndrome (AIDS) in earlier studies [100] and in forest rangers in our study. This finding provides evidence for the possible transmission of cryptosporidiosis between wild deer and persons in close contact with them. Importantly, *C. xiaoi*, *Cryptosporidium* sp. bamboo rat genotype I (formerly *C. ubiquitum*-like), and *Cryptosporidium* sp. bamboo rat genotype III (formerly *C. parvum*-like) were also identified in animals from the tropical wildlife park [62], suggesting that they may represent common species/genotypes circulating among diverse animal species in Hainan, and highlighting the potential for cross-species transmission within the island’s ecosystems. Of particular interest, many of the *Cryptosporidium* species/genotypes detected in this study, including bamboo rat genotype I, bamboo rat genotype III, and hamster genotype, are commonly associated with rodents. Notably, these genotypes have also been identified in other animal species in Hainan, such as sika deer, emus, and African lions [62], indicating that they may be widespread among animals in the Hainan region. The *Cryptosporidium* sp. hamster genotype was first identified in Siberian hamster (*Phodopus sungorus*) in 2009 [101] and was the predominant *Cryptosporidium* genotype circulating in pet hamsters in China [31]. One *Cryptosporidium* sp. hamster genotype isolate was identified in a wild Eld’s deer and another in a sympatric forest ranger. Moreover, based on *SSU* rRNA gene sequencing analysis, the two isolates showed 100% sequence homology. This is the first identification of *Cryptosporidium* sp. hamster genotype in cervids and humans, providing evidence for the potential zoonotic transmission of this genotype. However, it is important to note that, in this study, all forest rangers who participated, including those who tested positive for *Cryptosporidium* sp. hamster genotype and *C. xiaoi*, were asymptomatic at the time of sampling and reported no history of diarrhea or other clinical signs compatible with cryptosporidiosis within the past month. PCR detection in the absence of symptoms or microscopic confirmation of oocysts may indicate mechanical passage rather than true infection. Therefore, molecular detection alone cannot distinguish between active infection and transient environmental exposure. Future studies incorporating microscopic examination and longitudinal sampling are needed to clarify the clinical significance of these genotypes in humans. In our study, sequence analysis revealed that *Cryptosporidium* isolates harbored several genetic polymorphisms, including both SNPs and deletions. These nucleotide variations may reflect host-driven adaptive evolution and could be indicative of cross-species transmission potential for these species/genotypes. Although we took precautions to collect only the inner portion of fecal samples using sterile spoons to minimize contact with soil, environmental contamination from rodent activity in the sampling areas cannot be entirely excluded. Therefore, future studies should include environmental sampling (e.g., soil) and rodent feces collected from Eld’s deer habitats to better assess the role of environmental reservoirs in the transmission of these genotypes. Additionally, analysis of the 60 kDa glycoprotein (*gp*60) gene, the 70 kDa heat-shock protein (*hsp*70) gene, and the actin gene should be undertaken for the genetic characterization of different *Cryptosporidium* species, to confirm their specific identity and more accurately determine the risk of zoonotic transmission. These observations reinforce the need for sustained molecular surveillance at the wildlife–human interface, thereby facilitating the monitoring of emerging and spreading zoonotic *Cryptosporidium* variants.

*Giardia bovis* was the only *Giardia* spp. genotype in wild Eld’s deer. This species has also been detected in wild deer in Scotland [102], roe deer in Spain [54], and sika deer [13, 14, 62] and alpine musk deer [52, 57] in China. For *Giardia* spp., species display different degrees of host specificity, with *G. bovis* generally considered a hoofed livestock-specific genotype [103]; however, it has recently been described in humans in Australia [104], Brazil [105], and Egypt [106]. Although *Giardia* spp. were not identified in forest rangers within the protected area in our study, the zoonotic potential of *G. bovis* suggests a possible risk of transmission from wild Eld’s deer to humans. It has been reported that there was lower genetic differentiation among populations than within populations for *G. bovis* in artiodactyls in China, suggesting that this species may have undergone a population expansion [107]. In this study, several nucleotide mutations were also identified in the *G. bovis* sequences based on the conserved fragments of the *bg* and *gdh* genes. These nucleotide mutations may be associated with host adaptive evolution or geographic isolation characteristics of *Giardia* spp.

Co-infections with at least two species of protozoa are common in animals, such as bovines [81, 108], goats [109], dogs [110], and deer [102, 111, 112]. Although the present study did not detect co-infections with intestinal protozoa in humans, the co-infections were detected in 3.2% (7/217) of Eld’s deer, including four cases of *Blastocystis* sp. and *Giardia* spp., and three cases of *Blastocystis* sp. and *Cryptosporidium* spp. In our study, the occurrence of co-infections involving *Blastocystis* sp. may be attributed to its notably higher individual prevalence (25.8%, 56/217) compared with the other two protozoa, which increases the probability of its participation in mixed infections. Additionally, *Blastocystis* sp., exhibiting a positive association with other gut microbes, may act as a key facilitator due to this unique ecological behavior [113]. Its presence and potential immunomodulatory effects could alter the gut microenvironment, possibly increasing host susceptibility to concurrent colonization by more overtly pathogenic protozoa such as *Cryptosporidium* spp. and *Giardia* spp. The widespread distribution of *Blastocystis* sp. across diverse animal hosts, including wildlife, underscores its successful adaptation and persistence, further supporting its common occurrence in co-infection scenarios [114]. Notably, while the pathogenicity of *Blastocystis* sp. alone remains debated, its role in a polymicrobial context may be significant [115]. Co-infections could lead to additive or synergistic damage to the intestinal mucosa, potentially exacerbating the malabsorptive diarrhea typically caused by *Cryptosporidium* spp. or *Giardia* spp. [116]. This may translate to more severe clinical outcomes, such as prolonged diarrhea, greater weight loss, and compromised nutritional status, especially in juvenile, aged, or otherwise immunocompromised deer [117]. For endangered populations such as the wild Eld’s deer, chronic subclinical co-infections could act as a hidden stressor, reducing individual fitness and resilience to environmental challenges, thereby impacting overall population health dynamics.

## Conclusions

This is the first study to investigate the colonization frequency and molecular characterization of *Blastocystis* sp., *Cryptosporidium* spp., and *Giardia* spp., at the first time, as well as their zoonotic potential, in wild Eld’s deer and forest rangers working in a nature reserve in Hainan, China. The findings in the present study indicated high positive rates and rich subtypes diversity of *Blastocystis* sp. in Eld’s deer. The detection of shared *Cryptosporidium* species (particularly *C. xiaoi* and the *Cryptosporidium* sp. hamster genotype) between Eld’s deer and forest rangers provides evidence for potential zoonotic transmission at the wildlife–human interface. Further research is needed to clarify transmission pathways and assess the long-term impacts of these pathogens to evaluate their potential threats to public health, veterinary health, and Eld’s deer conservation.

## Data Availability

The relevant information has been included in the manuscript. The sequence data were submitted to the GenBank database under the accession numbers ON261379, ON261518, and ON261403 for *Blastocystis* sp.; PQ817700-PQ817702 for *Cryptosporidium* spp.; and PQ824566–PQ824570 for *Giardia* spp.
